# Under the bridge in Tehran: Addiction, Poverty and Capital

**DOI:** 10.1177/1466138118787534

**Published:** 2018-08-02

**Authors:** Maziyar Ghiabi

**Affiliations:** University of Oxford, UK

**Keywords:** ethnography, Iran, drugs, addiction, lumpen, city, homelessness, policing, Tehran, Global South

## Abstract

The article provides an ethnographic study of the lives of the ‘dangerous class’ of drug users based on fieldwork carried out among different drug using ‘communities’ in Tehran between 2012 and 2016. The primary objective is to articulate the presence of this category within modern Iran, its uses and its abuses in relation to the political. What drives the narration is not only the account of this lumpen, plebeian group vis à vis the state, but also the way power has affected their agency, their capacity to be present in the city, and how capital/power and the dangerous/lumpen life come to terms, to conflict, and to the production of new situations which affect urban life.

## Prologue. *To Burn, Burn, Burn* in Farahzad Valley

Out from their mothers’ womb

They found themselves on the walkway or in prehistoric

lawns, signed up in registries

that want them ignored by all history…

(Pier Paolo Pasolini, *La religione del mio tempo* [The Religion of My Time], 1959)

Under the highway bridges of Teheran’s ever-expanding urban landscape or in the alleys and backstreets of its popular neighbourhoods, a spectre is haunting the respectable middle-class and, with it, the state: the spectre of the ‘addict’.^1^ Riding to the end of a slope in a collective taxi, I get out of the car and ask for directions of a middle-aged woman with a loaf of *lavash* bread walking down the road. ‘Salam, *khasté nabashid*! Do you know where the *shelter* for homeless people is in Farahzad?’^2^ It takes a few moments for her to register the question. ‘Do you mean the addicts’ refuge [*panahgah-e mo’tadan*]? That one is at the very end of the road on your right-hand side, before the *darré* [valley]. Mind what you’re doing, it’s not a place to go.’ On my way I see several men, with worn-out clothes, picking up rubbish from the ground and stuffing it in huge fibre bags, which they carry on their backs. They seem to me the urban equivalent of the *nankhoshki*, the dry bread collectors that up until recently animated the morning wake-up of many Iranian towns. The men on this road do not use the rhythmic cry ‘*nunkhoshké, nunkhoshké*’ of the bread collectors. They do not collect dry bread for husbandry but the city’s waste to sustain a chronic dependence on heroin or methamphetamine (locally known as *shisheh*, ‘glass’).

These garbage collectors walk around furtively; their presence evidently bothers the neighbourhood residents, who belong to the white-collar middle class. Following this flow of collectors up the road, I end up in an open green space which is just south of the Emamzadeh Davoud, an old shrine in North Tehran. It is one of Tehran’s ancient *mahallé*, neighbourhoods, part of the Shemiranat district. The shrine hosts one of the countless descendants of the Shi’a Imams, who is thought to have arrived in the area with the Eighth Imam, Imam Reza, whose mausoleum sits in Mashhad, the capital of Khorasan, in North-Eastern Iran. So, on one side of the valley there is the shrine and on the other a shelter for homeless drug users. The body and the spirit of Tehranis find solace in their own ways.

Most of those frequenting this *patoq*, ‘hangout’, do not live in the neighbourhood, although there are locals who spend their evenings there (Figure 3). Better prices, steady availability and less variable quality guarantee a better consumer experience even in the face of the sheer degradation that the *patoq*’s setting showcases. The Farahzad Valley (*darreh-ye Farahzad*), where the shelter is located, has become a notorious site of drug use and open drug dealing in the last decade following the arrests and clampdowns in other areas of Tehran. Journalists have named it ‘The Autonomous Area of Farahzad’, while the police have attempted, for some time, unsuccessfully to bust the drug lord of Farahzad, Said Sahné, allegedly one of the biggest in town (*Tabnak*, 2013). Police operations, with the intention of clamping down on drug users’ gatherings in more visible areas of the city, drove flows of drug users to this traditional neighbourhood. In turn, the area became a successful market of narcotics and stimulant distributions, connecting the traditional crime-ridden districts of the south – notorious for drug dealing (as I describe later) – with the westernised bourgeois north.

**Figure 1.**
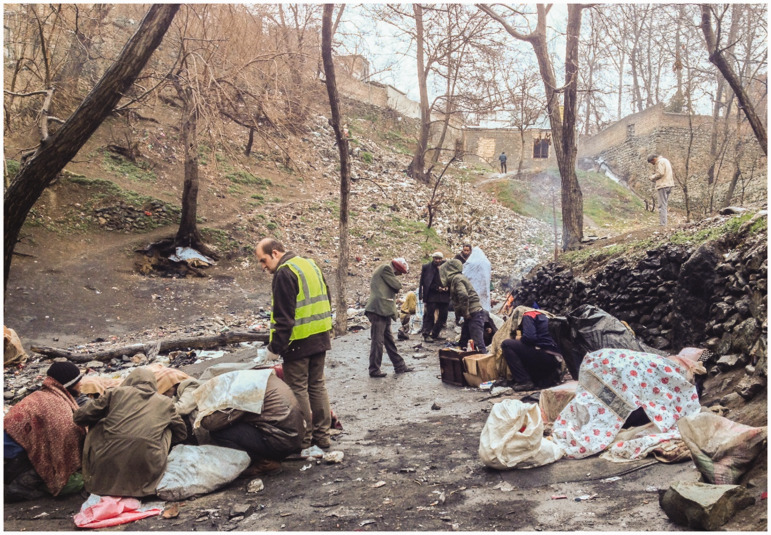
Chehel Pelleh. Photo by author.

**Figure 2.**
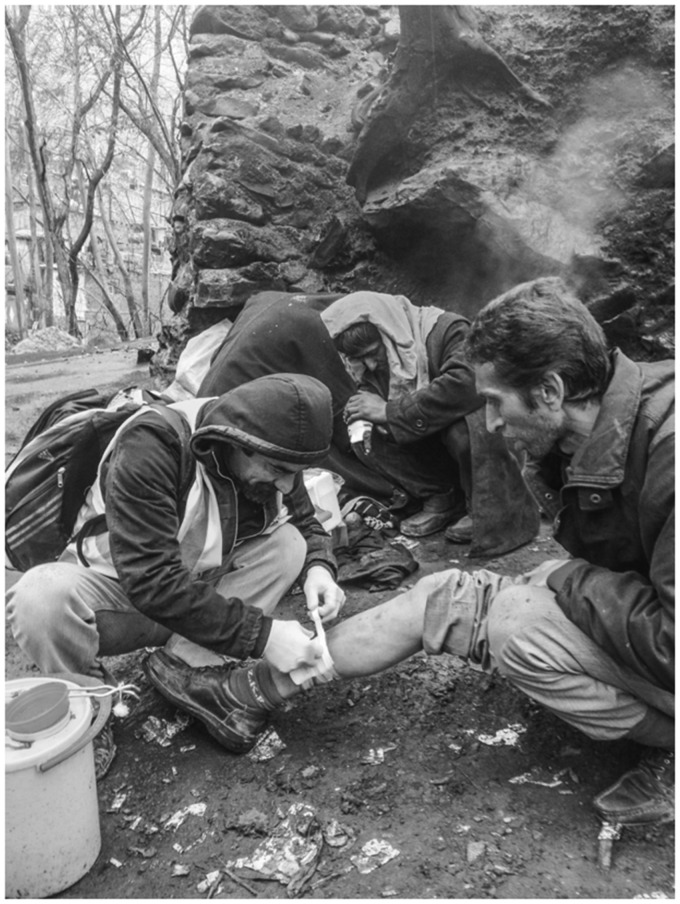
Harm reduction in Farahzad Valley. Photo by author.

Practically, Farahzad Valley’s drug scene is made up of numerous *patoqs* (Figure 1–2). The one closest to the shrine is *Chehel Pelleh*, the ‘Forty Steps’, which sits at the bottom of an old staircase – once made up of 40 steps, today mostly a remnant of bricks and clay. It is known as the biggest of all hotspots in the area. The bustling is continuous with some arriving from the main road from the closer residential complex and others descending from the shrine’s neighbourhood. Strategically located, the site remains insular from the main roads and, therefore, from the police. It operates in an economy of its own:

On flat ground surrounded by trees and streams of water lies the ‘high street’ of the *patoq*. A home-made wooden desk is placed on the muddy route, with a man dispensing different cuts of aluminium paper and various models of lighters. He sells the paraphernalia necessary for heroin smoking, for the modest price of one to two thousand *tuman* (ca. £0.50).^3^ On the other side, there is the *tarazudar*, the ‘weight-scaler man’ who is the person in charge of drug dealing, especially quantities exceeding the single dose. The *tarazudar* dispenses heroin, a substance in vogue in Tehran since the late 1950s (Ghiabi, 2018c), and methamphetamine (*shisheh*), the stimulant drug which had become popular since the mid-2000s. The main stash of drugs, I’m told, is secreted in several places by the local boss. In case of police raid, which is not an unforeseeable occurrence, the main dealer has the option of running away (e.g. motorbikes, hide-outs in the neighbourhood) or of hiding in the crowd of other drug users. In the latter scenario, he risks being arrested but avoids being recognised as a dealer. He steers clear of the risk of draconian penalties for dealing, which in Iran ultimately included execution up until 2017.

Security in the *patoq* is guaranteed by a number of *gardan kolofts*, ‘roughnecks’ who act as vigilantes in the drug hotspot. They consist of look-outs, informers, but also enforcers of the local order. They carry clubs and knifes but no gun, at least according to the widespread belief of those in the surroundings. The boss of the *patoq* pays them a daily wage for their services, mostly in the form of drugs, which they re-sell, or limited monetary compensation, or a combination of both. Strangers are dissuaded from passing through the *patoq* for fear of being taken for undercover cops or informants. This applies at times also to humanitarian groups, such as outreach activists who provide clean needles, condoms and primary healthcare to the people there. On one occasion, following an earlier police busting operation in the area, one of the vigilantes threw a heavy wooden club at me from uphill shouting ‘boro gomsho ****!’, ‘get lost, ****!’. Suspicion and distrust towards strangers is the rule, a fact that undermines also health initiatives among people using drugs, especially injectors and sex workers (Figure 2).

The reason why HIV prevention activities are often rejected was, in the words of one of the bosses, ‘because here in *Chehel Pellé*, we don’t have them! *Tazriqi nadarim*, we don’t have injecting drug users, we don’t want them, they are dirty. We are clean. Here is a clean space!’ Perplexed about his statement, he added: ‘Go to the *patoq* Mohammad Deraz, you can give this stuff [clean needles, etc.] there, *unà tazrighian*, they’re injectors!’ Yet HIV is rampant in these sites and mobility between different hotspots increases the chances of transmission of diseases, whether through shared injecting paraphernalia or sexual encounters. Keeping a low profile, many drug users would approach those distributing clean needles to be tested for HIV and hepatitis. They would usually walk away with half a dozen clean syringes and a few condoms.

Within the *patoqs*, there is a social/ethical stratification. About 20 minutes’ walk from *Chehel Pellé*, there is *Mohammad Deraz patoq* (Tall Mohammad), named after a local dealer who, according to the local myth, was very tall. The atmosphere in this *patoq* is more relaxed compared to that of *Chehel Pelleh*; there is a combination of people from different social backgrounds, men and women, and in general younger people. But not only young people frequent the enclave; it also functions as a tented encampment. It is there that I encountered Fereshteh and her boyfriend.

A young couple covered in dirt, with soot-streaked faces, sat in front of a small blue tent. I met them around 10am while they were smoking heroin on a piece of aluminium. ‘They had just woken up and were preparing their breakfast’ explained Hamid, the person who introduced me to them. Fereshteh welcomes me with a big smile which reveals the poor state of her teeth. Her lips are dark of the smoke of the opiate and her face swollen. She must be no older than 25, I thought. After the mutual presentations she offers me an apple, as per courtesy in Iranian culture – and regardless of the destitution in which she and everyone there lived, insists that I must have something while we sit together. So we split the apple in two and we eat it; her boyfriend declines to share it out of courtesy. ‘We have less than nothing and that is all I can offer you’, and taking up the aluminium Fereshteh says, ‘Not to be impolite: *befarma*! Help yourself if you please!’ I decline her kind offer of heroin, saying that I’m OK with the apple. She asks me whether I am a recovered addict, because ‘you look good’, ‘you’re healthy’ and ‘it’s evident you’re in a good state’. Then she adds, ‘Wow, I haven’t talked to a non-addict for such a long time, it is so nice to speak to people who are not addicts. Here everyone is an addict; everyone uses drugs or is in recovery or has been addicted. All NGO workers, too, are former addicts, they all hung out here with us up until recently [while nodding towards Hamid, the NGO worker].’ She chases the dragon, inhales it through a tube and concludes, ‘It’s been three years since I spoke to someone who is not an addict’.

Fereshteh has tried to ‘get clean’ several times. Earlier that year, she signed up in a methadone clinic, which happened to be near the shrine uphill, managed by a philanthropic doctor who provides free-of-charge services. She had felt awful, thought of dying and that is, she continues, because her drug of choice is not heroin – and she says so while holding some in her hand – but *shisheh* (meth). Methadone, the legal substitute for opiates, does not work for her; ‘I need energy and speed, otherwise I starve’. Her heroin use is to relax and have a sound sleep: ‘*mesl-e takh bekhabam*’, and take away her body pain: ‘*dardam bicharam kard*’. So she does heroin when she wakes up and before going to sleep. *Shisheh* keeps her going while at work and hustling. And, ‘for pleasure’.^4^

She has lived in the tent with her boyfriend for more than a year. Her look is that of a sick, dirty and dishevelled person. He sells ‘used’ stuff and stolen goods while she collects garbage and recycles old clothes. They are not married but they live together. Their existence parallels the ‘white marriages’ of many young urban couples who, not ready for the formalities and commitments (including financial and of housing) of *de jure* marriage, opt for informal arrangements, by living under the same roof. This option, nonetheless, remains illegal and repressed – haphazardly – by state authorities (*The Guardian*, 2015). For Fereshteh this is the least of her troubles. Her boyfriend sells used mobile phones and does petty drug dealing around town. He has been in and out of treatment centres, but with no success. Fereshteh collects garbage – one of the few female collectors I met – and, when desperate, she begs. ‘We want to get back our lives and return to society. Pray for us! Three years ago I used to study and go to school and now I’m in this…’. She is suddenly interrupted by her boyfriend, ‘Careful! You’re on fire!’. Fereshteh looks at me for a second or two, evidently on the heroin high, smiles and replies, while taking off her hood, ‘puff, if I were at home I would be screaming like crazy here and there. Here, it doesn’t matter, it’s like this, we live like this, it’s normal. We set ourselves on fire!’

Her hood had caught fire while she was lighting up some heroin. A few metres away, later in the day, I encounter an old man – or perhaps a 40-year-old who looks 70 – whose tent was burnt to ashes. His response, aware that all he had was now up in smoke, is emblematic of lumpen life on drugs, ‘It doesn’t matter, it’s a *sadagheye khoda*, a charity for God, it saved my life, if only I were there asleep in the tent now, I’d be dead.’ The old man and Fereshteh don’t care about burning their stuff to ashes, because, after all, life on the road is, to borrow from Jack Kerouac, *to burn, burn, burn* (Kerouac, 2002: 6).

## Vocabulary of Situations

Such is the situation at work in drug wars against the dangerous classes of the addicts. Drug wars, in the first place, take the form of powerful criminalisation of individuals’ drug consumption. Prohibition is the ideological frame that governs drug wars and that, ultimately, produces spaces of repression and isolation, in the forms of prisons or under the bridges of drug ghettos. These spaces of destitution with their local distinctions across the globe are bearers of similar semantic traits (Bourgois, 2003; Bourgois and Schonberg, 2009; Fernandes, 2002; Ghiabi, 2018a).

In Iran, addiction defines the poor categorically. In Tehran alone there are around 15,000 homeless people, with the number changing according to the seasonal migration from the hinterland. This migration is driven partly by the search for a living and the promise of higher revenues in the capital Tehran; but it is also the result of seasonal climate. The southern hinterland has a climate that is hardly tolerable for homeless and vagrant people in the summer, whereas Tehran, situated 1000m above sea level, has milder summer nights, which makes it ideal for open-air sleeping. This works also the other way round: Tehran’s snowy and cold winters compel large numbers of homeless vagrants to seek refuge in the milder climates of the southern plateau. The migration to Tehran signifies the arrival of low-skilled cheap labour employed in desultory tasks, whereas in the south this prospect is absent.

Overall, the number of homeless people addicted to drugs reaches 42,000 nationwide (*ADNA*, 2016), but this number is thought to be a conservative estimate. They are called *kartonkhab*, ‘the cardboard-box sleepers’, *velgard*, ‘the vagrant’, *bikhaneman*, ‘the homeless’, but the category through which they are governed is what the police has named *mo‘tadan-e porkhatar*, ‘the dangerous addicts’. In fact, more than 80 per cent of the homeless population is, according to official sources (Mehr, 2016), addicted to illicit substances: heroin, meth, alcohol, morphine, methadone and so on. A high incidence of HIV/AIDS (a blood-borne disease easily spread by sharing injecting equipment) and sexually transmitted diseases (STD) have also been revealed, despite this population remaining largely invisible or hidden in the cityscape. Its ecology of existence is made up of the popular districts of the south, the valleys and caves in the north, the undeveloped lands under the highway bridges and, recently, the graveyards in the suburbs (Habibi and Ghiabi, 2017).

Following the opening ethnographic scene, the article provides an ethnographic account of the lives the ‘dangerous class’ of street addicts based on fieldwork carried out among different drug using ‘communities’ in Tehran between 2012 and 2016. It explores the themes highlighted in the opening ethnographic scene, i.e. the place of homeless (dangerous) drug users; the structural violence of their lives; and the political economy of lumpen existence. The primary objective is to articulate the presence of this category within modern urban life, its uses and its abuses in relation to power. What drives the narration is not only the account of this lumpen, plebeian group vis-à-vis the state, but also the way power has affected their agency, their capacity to be present in the city, and how capital/power and the dangerous/lumpen life come to terms, to conflict, and to the production of new situations which affect urban life. The article also tackles the theoretical and historical frame of reference upon which the narrative is built. This includes a glimpse at the history of drug prohibition, its rationale and its connection to the category of class, especially to that of lumpenproletariat and the dangerous poor. In this attempt, one can bridge the hermeneutic gap in the study of the non-western world, where the category of ‘dangerous class’ was first enunciated. It connects the lives of cities across the Global North and South, through in-depth description of lumpen situations the reader is invited to ‘see’ how categories born out of sociological analysis in other times and spaces can be at work, productively, in new contexts. The intent is to add not simply to the knowledge of new historical, ethnographic cases, but to enrich the understanding of the category of the ‘lumpen’ and dangerous class.

My encounter with the individuals described in this article occurred during an extended fieldwork project carried out in the city of Tehran. Comprehensive and synthetic elements of the experience of homeless drug users is narrated through dense ethnographic cases (Javad, Fereshteh, Mohsen, Hamid, Reza and Ali), built on personal immersion in their life settings, including dozen visits to the *patoqs*, ‘deep hanging out’ (Geertz, 1998) in the public and casual but sustained chats while strolling in the city or sat in proximity of their ‘private’ dwellings. Observation of the setting landscapes and the way it was transformed in the passing of time (from 2012 to 2016) also informed my ethnographic analysis. Although I am aware this is not exhaustive – as all generalisation betrays ethnographic details – the description of events, settings and modes of existence mirror recurring traits in the drug-using communities I had the chance to study during my fieldwork. Contacts with individuals occurred also while I volunteered with harm reduction outreach programmes in Tehran. Meetings occurred both during outreach programmes and outside those settings.^5^

## The structural violence of lumpen life

The lives of lumpen drug users have become the object of structural violence produced by capitalist forms of government and exploitation (Singer and Page, 2016: 153; Bourgois and Schonberg, 2009; Bourgois, 2003). This violence is both physical and symbolic as it operates through mass incarceration of minorities and the marginal, police killings and large-scale substance abuse as well as violence against these communities through the ideology of ‘individual achievement and free market efficiency’ (Bourgois, 2011: 7; Garcia, 2010). While spatial segregation, in the forms of prison or the poor people’s ghetto, impedes human development through the exclusion of the working class and the undeserving poor – like homeless drug users – from the mainstream economy, ideological exclusion condemns them to a virtual oblivion. Communities depleted by the addiction and anti-narcotic assemblage, the duo of chronic health shortcoming and selective judicial and police targeting, go through a process of lumpenisation, which goes hand-in-hand with the depoliticisation of unruly subjects in the city. Criminal records and spatial confinement to urban neighbourhoods reputed as unhealthy and criminal become thus a structural obstacle for seeking employment; drug offences make that task a virtually impossible mission. A general look at the scholarly literature cited above shows that this process is one of lumpenisation of plebeian classes and, from a structural viewpoint, does not differ across the East and West, North and South divide (Bourgois, 2018; Ghiabi, 2018b).

One cannot speak of ‘class’ in reference to drug users. The category of drug user itself is an invention of a political machine which has listed certain substances and plants as exceptional (poppy/opium, coca/cocaine, cannabis/marijuana for instance), but regulated others (tobacco, alcohol) in a flourishing capitalist market. This machine emerged in the first part of the 20th century concomitant with state-led modernisation and through the influence of US anti-narcotic discourse – though countries in the Global South and, especially, in the Middle East and North Africa (MENA) developed their own prohibitionist vision of the drug wars (Ghiabi, 2018e, Ghiabi et al., 2018). The drug ‘addict’ – to use the lexicon of 20th-century drug wars – is neither part of the economic system of production nor of its moral order. He or she is a parasite *par excellence*, because in the popular imagination he/she remains useless – of no use. He/she relies on illegal income or on charity and is therefore a liability in the political economy of development. Yet, chronic drug users – aka ‘addicts’ – are also avid consumers, the nature of drug use being tied with chronic drug consumerism and the unending search for money/capital. This inescapable drive for consumption is what makes chronic drug users an essential identity/product of capitalist times. Therefore, ‘addicts’ are not a class, but a category that cuts across social classes and is instrumental in discriminating against the poor and the ethically underserving.

The experience of living under drug prohibition brings the wage labourers of factories, farms and industries closer to the life of the wageless, the so-called people of the ‘informal economy’. That levels the ground between proletariat and lumpenproletariat. Michael Denning argues that ‘we must insist that “proletarian” is not synonym for “wage labourer” but for dispossession, expropriation and radical dependence on the market’ (Denning, 2010: 81), including the illegal drug market. Dependence on the market defines the life of drug users in structural ways. The wage labourer, in parallel, under a system where those consuming certain substances are objects of police repression, becomes what Marx called ‘a virtual pauper’ (Marx, 1993, cited in Denning, 2010) for whom poverty is a reality in waiting, likely but not necessarily inescapable.

Under this rationale, the figure of the ‘addict’ – its etymology reminding us of its Latin root, *addicere*, ‘to enslave’, and particularly that of the homeless, street drug user – establishes itself as a preeminent example of a class in dependency under capitalist production; a class dangerous for its nature threatens bourgeois decorum and in danger because it is at the mercy of class-based subjugation. Hence comes the making of poor drug users into a ‘dangerous class’. Programmatically perceived as a bearer of danger, disorder and irrational violence, its danger is manifested through different concerns, which encompass and enmesh criminality, health, morality and middle-class prosperity – elements that emerged in the opening ethnographic description. The danger, however, is binary: seen as socially dangerous and political/ethical misfits, these are also categories in danger, because their life – qualifying as ‘bare life’ (*zoë* as opposed to *bios*; Agamben, 2010) – is disposable, precarious, wasteful, contaminated. Their death, too, is *bare*, as it does not leave public signs beyond statistical records (cf. Comaroff, 2007; Biehl, 2013).

This overlapping of medical, sociological, criminologist and political concerns is a side-effect of the medicalisation of politics vis-à-vis the categories of the deviant. This process had among its founders the Italian physician (and ‘physiognomist’) Cesare Lombroso, who first sought to preserve, on shaky medical grounds, the healthy from the fool and the revolutionary (‘*i mattoidi e rivoluzionari*’) (Lombroso, 1896), an expedient to preserve the status quo and middle-class decency amidst plebeian revolts of 19th-century Europe. Dismissed by scientific knowledge, his theories confuted, their implication was maintained in the nexus between medicine and criminology of which drugs policy is an especial field (though I am not aware of Lombroso’s direct influence on Iranian criminologists). In this medico-political frame, medical understanding enables a moral and political judgement, which bestows upon scientific definitions (such as mental disorder, addiction, etc.) a clear classist character. The rigid categories of medical sciences enmesh with the more ambiguous ones of human knowledge, in a classist plot clothed in a neutral, *technical* language. The excitement of criminological reactions from the state vis-à-vis the homeless drug users is, in the words of British psychiatrist R.D. Laing, ‘a social fact which in turn it is a political event’ (cited in Basaglia, 1971: 168). I shall now dwell more closely on the structural violence which has as its object the dangerous class of drug addicts.

Over the decades following the Islamic Revolution in 1979, the authorities referred to unruly members of the suburbs with the derogatory term *arazel va owbash*. Its meaning is vague, but anthropologist Shahram Khosravi suggests that, since 2007, it has become a notion constructed in opposition to the romanticised image of the *louti*, the gallant delinquent of the traditional neighbourhoods surrounding the bazaar (2017: 104–5; Adelkhah, 2000). The etymology of *arazel* can be traced back to the Arabic root ‘R-DH-L’, which indicates something ‘low’, ‘abject’; *awbash* instead stands for ‘riffraff’. Both are of Koranic derivation and of utmost negative value. Updating the Marxist use in *The Eighteenth Brumaire of Louis Napoleon* (2007 [1982]), of ‘social scum’, ‘rotting mass’ and ‘disintegrated mass’, *arazel va awbash* can be synthetically translated as ‘*lumpen*’.

The law enforcement and political cadres of the Islamic Republic remained vague on who exactly belonged to this multifarious group of dangerous individuals. Those arrested for being *arazel va awbash* were systematically accused also of being involved in drug dealing and/or being addicted. In the official discourse, being connected to drugs triggered an association with a milieu of sexual depravity, moral decadence, alcoholism, Satanism and *zurguyi*, ‘bullying’. The danger posed by them is that of destabilising the moral order on which the Islamic Republic rests its legitimacy. The lumpen people, unable to adapt to the engaged and moderniser momentum of the post-war era (post-1989), risk contaminating its body politic. From within they contaminate through physical and psychological deviancy: addiction. From without, they contaminate as part of an ‘imperialist plot’ (*toute’ este‘mari*) aimed at undermining the Islamic Revolution, through the diffusion of the westernised decadent lifestyle.

Since the early days of the 1979 revolution, addicted people have never been regarded as deserving the compassion of the Islamist state. Rather than a result of Islamic tenets and scriptures, the lack of compassion for drug ‘addicts’ stemmed from secular moral considerations rooted in the anti-drug ideology of the early 20th century. Indeed, large-scale criminalisation had already been operating under the Pahlavi Monarchy (1925–79), justified by the need for social and cultural modernisation (Ghiabi, 2018e). With the Islamic Revolution, anti-drug campaigns acquired the terminology of anti-imperialism – drugs as an immoral western import – and of moral cleansing of the newly revolutionary social body. Drug users’ psyche and body were deemed at odds with the moral purity praised by the clergy. That is why, during the 1980s, treatment of drug abusers was not publicly available, since the official state strategy on addiction called for forced detoxification through incarceration and forced labour (Ghiabi, 2015).The targeting of plebeian classes is a development emerging out of the shift in governing cadres and their political economy after the end of the Iran-Iraq War (1980–88).

During the first years of the revolution, when Ayatollah Khomeini’s authority remained unchallenged, the *mosta’zafin* (the Koranic term indicating the ‘disinherited’) was used in opposition to the capitalist, westernised class of the *mostakbarin* (‘the arrogant’). Policies in favour of the poor and the urban proletariat were legitimised as part of the promise of social revolution to which many had pledged in 1979, amidst the revolutionary fervour (Bayat, 1994). The disinherited classes were directly inspired by Franz Fanon’s incitement to the *Les Damnés de la Terre* (*The Wretched of the Earth*, 1961), mediated through the work of Iranian revolutionary intellectual Ali Shariati, who supported a sui generis Islamic Liberation Theology. This legacy lasted up until the mid-1990s, when the necessities of reconstruction implied shelving the populist love for the disinherited in favour of developmental goals through investment and capital accumulation. Today, governmental cadres are pledged to an implicit taboo on the use of populist categories such as the *mosta‘zafin*.

Large-scale developmental programmes turned the capital Tehran into the centre of economic gravity, a process that speeded up the already mass urbanisation caused by war displacement in the 1980s. The face of the drug phenomenon had also mutated between the late 1990s and early 2000s, when HIV/AIDS, caused by rising heroin injection, had become a material threat to the general population (Christensen, 2011; Ghiabi, 2018a). A vast system of public health services had been put in place for people seeking medical assistance in kicking their habit (Ghiabi, 2018e). This shift towards a medicalised and more tolerant policy vis-a-vis drug consumption and drug dependence has had ambivalent outcomes. Middle-class drug users are provided with the choice of subsidised methadone treatment or an array of in-patient centres which differ in methods and philosophy with regard to ‘getting clean’. Drug users that have not the benefit of choice and, particularly, homeless and vagrant drug users are situated in a zone in between punishment and care (Ghiabi, 2018a), which highlights the structural violence which governs their existences. *Javad Sorkhé*’s (Red Javad) tale is testimony to this condition in the Iranian setting.

### A Javadi history of drugs^6^

Javad is a 32-year-old man. Born in Islamshahr, on the outskirts of Tehran, his family left their village in the Central Region (*Ostan-e Markazi*) to move to Tehran in the early 1970s. He is the oldest of five brothers and two sisters. Dropping out of school during his teens, he has worked as a taxi driver in Shahr-e Rey, bus driver assistant on the Tehran-Qom line, and petty drug dealer over the last 15 years. Opium was a formative element in his family life. His father and mother were both heavy opium users and by the age of 16 Javad himself had acquired a taste for opium, hashish and later, with his companions, heroin. His story is reminiscent of that of many young men in the lumpen city. Unemployment, illegal employment, family crisis, prison, disease, violence and drug abuse. All of these, he reiterates, impeded Javad’s attempt to get married: ‘no girl wanted the son of an addict’, ‘no girl wants someone without a future’, ‘all the girls look out for the rich kids’, ‘if you marry someone from here [the neighbourhood] it ought to be from a desperate family; I got enough desperation myself’. The death of his father put further pressure on him to sustain the family: ‘it was enough *for our hand to reach our mouth*, but drugs need money and we, *mashallah*, are all [drug] consumers!’

**Figure 3.**
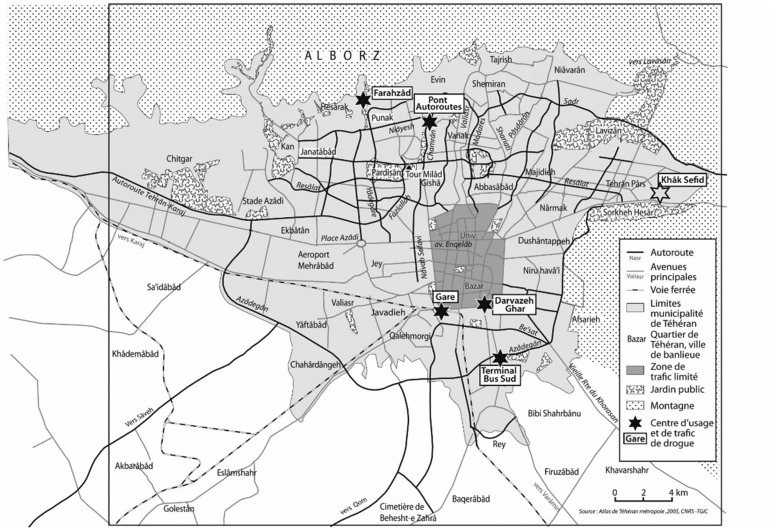
Map of Tehran’s drug hotspots. Source: Ghiabi, 2018d.

Unable to do so through the legal economy, which remained stagnant for unskilled manual labourers facing the cheaper labour of Afghan migrants who arrived in the 1980s, he is thrown into the informal market of smuggled goods and petty dealing: ‘I had a job, it was working out, then I got in some troubles with the man running the business and I was kicked out; then I got another job but *hammash khomar budam*, I was always high, couldn’t get my shit together’, ‘it was dangerous but I made enough to have my stuff and help my mom’. After a couple of years, ‘I got caught because some of the kids gave my name to get one year instead of five, that’s how it works’. On this line, he cites *Hich Kas*, the Iranian rapper he knows I listen to too, in the song *Ekhtelaf* [Difference/Disagreement/Conflict]: ‘*inja jangalé, bokhor ta khordé nashi*’ [Here is the jungle, eat not to be eaten!]. His words triggered in my mind the song’s following two lines, which I thought described the situation more closely: ‘*Ekhtelaf-e tabaqati inja bi-dad mikoné, bu-ye mardom-e zakhmi adamo bimar mikoné*’ [Class difference oppresses this place, the smell of wounded people sickens].

During the period I met Javad, he smoked heroin and *shisheh*. The first is a downer (opiate), the other a powerful stimulant, an upper. His meth habit produced great mobility in the city, new encounters, more efficient work/rest balance, but also increased paranoia and volatile relationships. His reactions to ordinary events, he confessed, had become more aggressive and he had lost the capacity ‘to wait’ (cf. Khosravi, 2017). Javad would purchase a few *suts* (1/10 of a gramme) and smoke it while on a stroll around town. In his own way, Javad is a plebeian *flâneur* of the modern city, his existence *being* present in the city mass but differently from his 19th-century equivalents, the 21st-century *flâneur* is speedy and is no gentleman.^7^ Unproductive, unemployed, he has nonetheless unparalleled knowledge of urban life and landscape, whereas the *flanerie* of the wealthy rolls exclusively on the wheels of fancy cars. Moving from South to North, by collective taxis, scooters, metro and walking, the presence of plebeian *flâneurs* is increasingly visible in modern Tehran (Figure 3).

It would not be unusual for Javad to stop on a pedestrian bridge overlooking one of Tehran’s highways. He would take out his glass pipe and torch lighter, inhale with a deep breath, holding in, blowing out, before putting everything back in his pocket. In one occasion, he confessed: ‘My body is used to morphine, *tarkib-am morfini-é*, my structure is morphine-like but this [*shisheh*] turns on my brain. Otherwise I’m lost’. With meth, he felt motivated to stroll across the city, beyond his usual neighbourhood, wandering. On the back seat of his friends’ motorbikes, on public services, he and his friends reasserted their presence in areas of Tehran outside their class-based domain. The unremarkable heroin smoker who moved his body only in order to hustle enough to keep his habit going had now become a remarkable urban presence. Accounts in the newspapers about *shisheh* smokers acting volatilely across the capital mushroomed throughout the 2010s (Khabaronline, 2014; Jam-e Jam, 2017; ISNA, 2014).

Javad was sentenced to prison on several occasions. Twice for petty dealing, once for thuggery. Meanwhile he developed a taste for heroin, which he smoked with the kids (*bacche-ha*), while killing time in southern Tehran. In prison, he needed heroin to avoid the heavy withdrawal symptoms, so he reverted to sharing heroin with other inmates through a self-made pump or in signing up on methadone substitution programmes. ‘Thank God, I did not get AIDS! But I had no other solution there’, he confesses while we walk around Harandi Park. Once out of prison he failed to find a stable dwelling and spent time wandering from parks to friends’ flats to the compulsory treatment camps to public methadone clinics. Treatment came always against his will following police arrests in one of the collection plans against drug addicts and the *arazel*.

I ask: ‘Why did the police arrest you? They keep saying “addiction is a medical issue”, “the addict is a person with a disease”, “we provide treatment for the addict” and then someone like you gets caught every two months.’ His response was telling of lumpen awareness of structural drug violence:

You know how the police work. Every now and then, the commander of some police unit decides that the statistics of crime are low, so the police captain comes to the office and says, ‘today I want 100 criminals’. So the easiest way to get this number is to raid a *patoq* and you can get as many as you like. They do you the [addiction] test and then send you to a [treatment] camp for one, two, three months… The addict is easy to get, so you know it’s convenient for them. Newspapers talk about us. The rich feel more secure. The shop owners sell more. Treatment centres get their subsidies. Even the dealers take a break so the price goes up. And us? *Khob*, well, *we* get fucked!

Clear evidence of the systematic use of the addicts as a useful expedient to increase policing records are difficult to obtain. But a quick look at the number of people incarcerated or referred to state-run treatment centres (mandatory treatment) over the last decades provides the reader with a telling picture. In 1989, the number of drug offenders in prison totalled c. 60,000. Ten years later it had reach 210,000 and in 2001 around 250,000.^8^ This steady increase of arrests is only partly justified by the more efficient anti-narcotics enforcement of the police. Javad’s arrest following his last prison term did not entail a specific crime, except for being a ‘dangerous addict’, i.e. living at street level. He wasn’t arrested for dealing drugs or smuggling illicit goods. He recollects being taken to a mandatory treatment camp, the notoriously violent *Shafaq* camp (Ghiabi, 2018a), at least on four occasions. ‘You get arrested together with a hundred other people and you end up in a place where you “detoxify” for a few weeks. Some people are happy about this especially when it gets colder in December and January. A warm place to stay for a few weeks and then you can get back at your place, in the park, anywhere, after Nouruz [the Iranian new year on 21 March]’, when the weather is milder. His account is not isolated and I had confirmation of this in the stories of other individuals.

Reports of homeless drug users – or simply vagrant paupers – freezing to death in Tehran’s cold winters are not sporadic. While carrying out fieldwork in the Farahzad *patoq*, there were several reports of homeless users falling asleep in the valley and freezing to death. Dying in a *patoq* does not carry a public signature – the vagrant’s death is an event with no sign – except for statistical data on drug deaths. It is a circumstantial event accompanying the journey from the *bare life* of the homeless addict to their *bare death* (Comaroff, 2007: 197), bare inasmuch it does not leave a trace – no signature – in public life. The overlapping of life and death in the existence of lumpen drug users is considered a consequence of the illegibility of this category: not fitting in the scheme of state-society life, their existence and non-existence blur into each other. Many homeless drug users, especially when belonging to far-away rural regions or to ethnic minorities including that of Afghan undocumented migrants, lack proper identification. Inability to provide the ID card means finding oneself in a limbo vis-à-vis the most basic needs, such as health, education, housing and welfare. It is the case of the children of many foreign fathers (especially Afghans), but also to many street drug users who amidst the unsettledness of their existence have lost track of their papers. With no identification, treatment/incarceration can be extended for longer periods.

The homeless, *bi-khaneh* or *kartonkhab*, exemplifies a case of *khanemansuzi*, a Persian expression used to describe the impact of drugs on people’s lives. It means ‘burning one’s house down’ and it captures the status of the homeless drug users beyond the metaphor. It suggests the abandonment in which homeless users exist, disconnected from family (which in the Iranian context is the foundational ethical site for social integration). The drug user, who has lost his/her dwelling, is equivalent to a *sans papiers*, someone who cannot be recognised, whose identity is troubling. In order to exist, he/she must pursue a life outside formal order to avoid being incarcerated or deported. To follow on the metaphor of *khanemansuzi*, the dwellings of street addicts become the *kharabat*, the ‘ruins’, the image used in Persian poetry to describe the wine tavern, the bandits’ nest, the sacred refuge, the utmost disrupted site of the soul.

Metaphors are abundant in lumpen life and they often bear empirical value. ‘We are in *Barzakh*’ Hamid tells me, another ‘experienced’ (his word) drug user from Darvazeh Ghar. ‘They take us, set us free, re-take us, it’s like a game’. *Barzakh* indicates the Islamic Limbo, the place where men and women wait at the end of time before God’s judgement. The Limbo, however, has its material grounds or, to put it crudely, *graves*. In November 2016, a shocking report in *Shahrvand*, showed groups of more than 50 homeless drug users living and sleeping in the graveyards of Nasirabad, on the Tehran-Saveh highway. Gathered in this cemetery, with piles of cartons, plastic bags and wood, they occupied around 20 graves. Two to four people live in each of the sepulchres where they burn wood to warm up in the freezing temperatures of the Iranian plateau. *Gurkhabi*, ‘sleeping in graves’, became the new public trope which caused the piety and compassion of social media users. The Oscar-winning director Asghar Farhadi even wrote a letter to President Hassan Rouhani, demanding that the government intervene with urgency.

**Figure 4.**
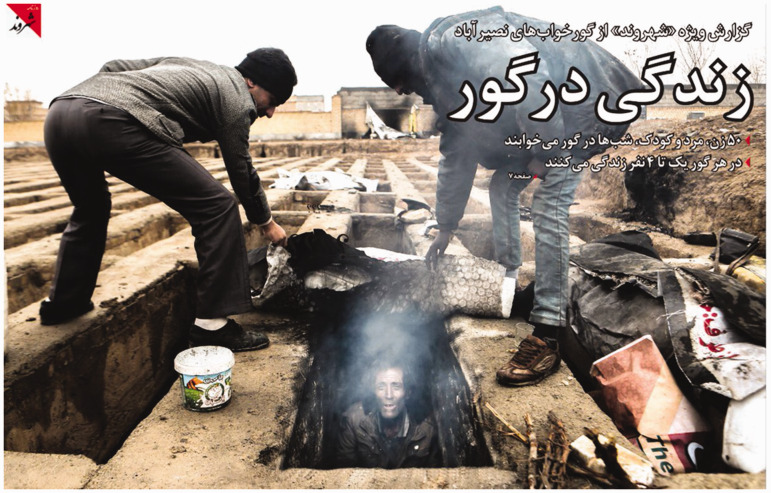
Front-page of Shahrvand: ‘Life in the Grave’.

Living in graves is indeed a powerful image in the public eye (Figure 4). Homeless users turn sacred, spiritual places, such as the shrine in Farahzad described in the opening section, into profane surroundings of human decadence. The cemetery too is stripped of its quiet and meditative dimension where ordinary people bring their sorrows. It is now a place of destitution of lumpen lives, the graves of the dead inhabited by precarious addicts. Yet, graves are safer and warmer places, ‘to avoid policing and the cold’, as a man in treatment in Shahr-e Rey explained. This is not an Iranian oddity. In Cairo, for instance, the dwellers in the *ashawiyyat*, the informal residences in Cairo’s huge cemetery, enjoy better conditions than those who live in the city’s crowded suburbs. Access to facilities, better connection to the city and insularity from state encroachment guarantee a safer existence (Bayat, 1997). Although the ‘gravesleepers of Nasirabad’ did not establish informal settlements like their Cairene equivalents, they too found themselves in a precarious, but safer, condition than those other homeless users in the parks or under the bridges. Following the report of grave sleeping, the police intervened in the cemetery and rounded up all the homeless drug users residing there. They were taken to a compulsory treatment camp. Once released, almost all of them continued using drugs. Their new residence, in many cases, became the surrounding deserts of Nasirabad (*Vaqaye‘ Ettefaqiyeh*, 2017).

## Structure and agency in the lumpen economy

Objects of a systemic violence that denies them a place to exist, homeless drug users live in a political economy of their own, made up of charity, hustling and sharing. The centre of gravity of philanthropic endeavours for the urban proletariat and homeless drug users is Darvazeh Ghar. Its name – which in Persian has a mysterious allure: *ghar* means ‘cave’ – is said to derive from an episode in which one of the sons of the Imam Musa al-Kazem, the seventh Shi’a Imam, while fleeing the officials of the government, took refuge in a cave and, as is often the case with Shi’a leaders, disappeared (Masjed Jamei, 2016). Over the 20th century, informal settlements dug in the ground and called *gowd* represented the most populated quarters of the area. The *gowds* hosted mostly brick makers who, given the lack of housing land, created their homes by digging in the ground. By the 1990s, under the Rafsanjani government, the *gowds* had disappeared, making way for a new urban project. The *gowd-e ma‘sumi* became Harandi Park; *gowd-e arab-ha*, Baharan Garden; *gowd-e anvari*, Khajavi Kermani Park; and *gowd-e Khalu Qanbar* was replaced by Haqqani Park.

**Figure 5.**
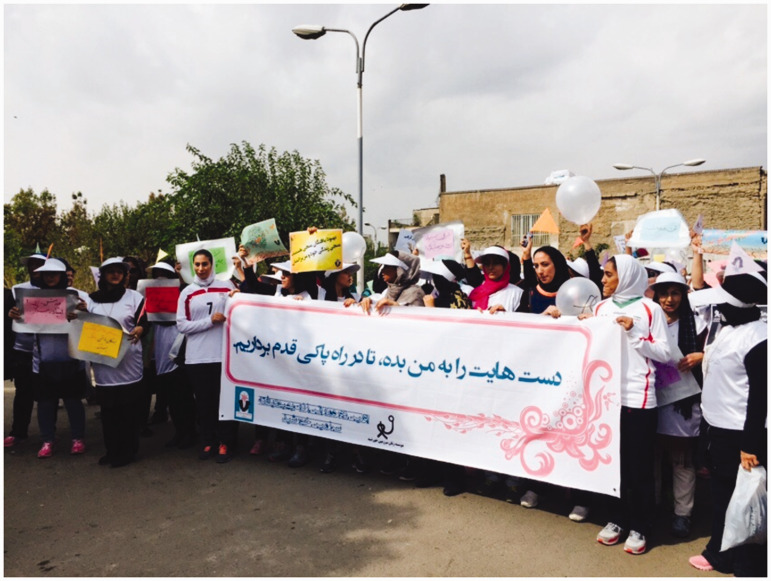
‘Marathon’ in Harandi Park. Photo by author.

**Figure 6.**
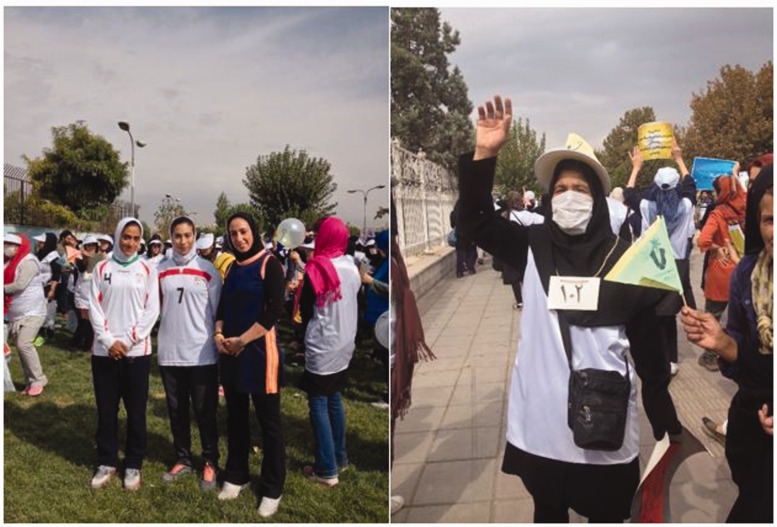
Left: football players from the Iranian National Team; right: a former homeless addict chanting at the marathon. Photo by author.

**Figure 7.**
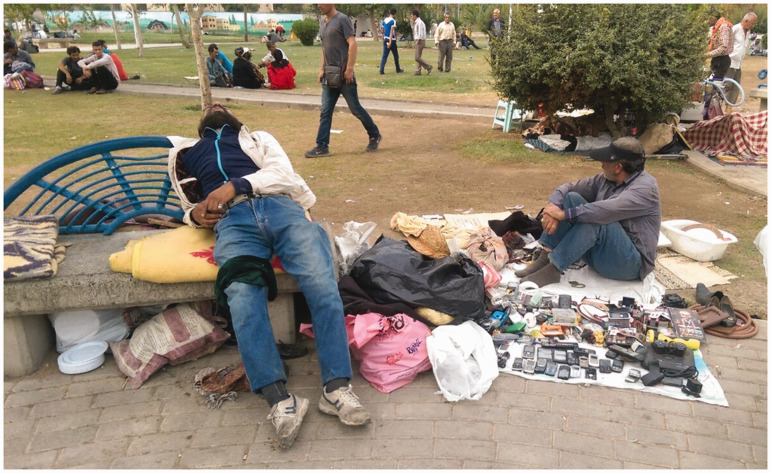
Harandi Park: street vendors, street addicts. Photo by author.

The four parks together are the centre of gravity of lumpen drug use in Tehran (Figure 5, 6 and 7). The parks and green spaces, carefully promoted by the Tehran municipality, have become dwellings for homeless drug users – hence ‘no-go zones’ for children and families. Shops and trades in these areas protest against their presence, which transforms ‘decent neighbourhoods’ into lumpen citadels. ‘We don’t sell anything except *nakh ‘oqabi* [one fag of *Winston Red*, popular brand for homeless and hashish smokers]’ laments a shop owner in Darvazeh Ghar. He adds ‘Even mothers who come here buy just half a kilo of lentils; no yogurt, no milk. Here, *harf-e avvalo e‘tiyad dare*! [Here, addiction runs the place!]’.

The genealogic presence of lumpen life is a durable trait of the neighbourhood, which in recent years has also seen large philanthropic activities. The presence of civil society groups had in fact become central in this area and public attention had reached its zenith when in autumn 2015 several groups of volunteers, humanitarian groups and philanthropic citizens started to bring cooked meals and clothes to the park and distributed them among the drug users. I happened to be in the neighbourhood during a few of these instances. Well-dressed women who attended charitable events taking place in the area would often take large pots of rice and stew and distribute them in the park. The courtesy, as it happened, provoked skirmishes and fights among the numerous homeless people in the park, who attempted to secure a warm, often sophisticated, Persian dish. Some of the women remained baffled by the violent scenes and would soon walk – if not run – out of the park. Despite the incident, the area witnessed a steady increase in charitable work. A few months later, a charity organisation started to paint across Iranian cities, starting from Tehran, ‘walls of kindness’ (*divar-e mehrabani*), encouraging Tehranis – and later all fellow Iranians – to bring warm clothes, food and other essential items for those in need.

The provision of food and clothes had become also a matter of satire. Detractors hold that ‘the drug addicts are no longer satisfied by bread and egg or bread and cheese, but they expect sophisticated food and are spoiled for choice’ (*Sharq*, 2015). Others claimed that public attention is driven by a sentimental piety not grounded in a real understanding of the complex situation of drug addiction, especially in the Harandi area. In the words reported by a piece on *Sharq* (2015), this humanitarian approach was a type of ‘addict-nurturing [*mo‘tadparvari*]’. Another public official cynically suggested that the provision of food might well be a stratagem used by providers of addiction treatment centres to attract people to their facilities and, incidentally, attract public funding to their organisations.

On 9 October 2015 I was invited to attend the ‘First Marathon of Recovered Female Drug Addicts’ organised by the House of Sun (*khaneh-ye khorshid*), an event which took place across the four parks of Harandi, Razi, Baharan and Shush (Figure 5 and 6). On the edge of Harandi Park’s southern corner, the House of Sun has been active for over two decades in providing free-of-charge services and support to female drug users and those women seeking refuge. A large crowd of women (and some men) attended the opening ceremony of the marathon and waited for the start of this seemingly sporting event. Two female players of the Iranian national football team led a collective session of gymnastic activities, a way to symbolically recover the body of the park from the sight of widespread drug use and destitution. Truth be told, the event revealed itself to be not a marathon – not even close – but rather a public demonstration that brought more than a thousand women and their sympathetic supporters (like myself) to march inside the park and in the middle of the gathering of drug users, among whom there were dozens of women. The term ‘marathon’, I thought, was probably used to get around the politicisation of the event in the eyes of the municipality, which could have regarded a women-led march against drugs as too sensitive a topic.

Leila Arshad (aka Lily), the main organiser of the event and director of the House of Sun, had long been working in this neighbourhood. While those attending the marathon had gathered in the courtyard of the NGO, she held the microphone and said, ‘one of our objectives is to catch the attention of the public officials and people towards your problems: lack of employment, absent housing, insurance and treatment, respect and social inclusion’. A few weeks following the marathon, a group of 40 men raided the informal camp in Harandi Park, set on fire several tents, and attacked a number of street drug users with sticks and clubs. The municipality declared that the attack was perpetrated ‘by the people’, denying any responsibility. Others hinted at the lack of responsiveness of the police (*Etemad-e Melli*, 2015). Notwithstanding this occurrence, the attitude of Iranians towards charity has changed significantly over the years. Mendicancy, begging and petty vending is accepted less *for God’s sake* and more in exchange for merchandise or services (Asfari, 2016). Philanthropy itself has transmuted and has lost its Islamic framework and become more market-oriented. The observation comes to mind of Italian psychiatrist (and reformer) Franco Basaglia who, while visiting New York, noticed on the metro line an advertisement: ‘Which of these human tragedies do you prefer? Vietnam, Biafra, the Arab-Israeli controversy, the black ghettos, hunger in India …? Choose yours and help, helping the Red Cross’ (Basaglia, 1971: 71). Despite the rise of philanthropy in favour of homeless drug users, the economy of lumpen drug users does not rely on charity exclusively. To get by, most street users find ways of making a living in creative and painful ways, as the case of Reza epitomises.

Reza comes from a well-off family, but he was expelled from his wife’s house because he was addicted to morphine. He now lives in a small room rented in the south of Tehran, where he carries on his morphine use, plus smoking meth. Recently he signed up to a methadone substitution programme and he is now trying to get off both drugs. He speaks frankly with me (while I often found him lying to the NGO workers with whom he sometime volunteers): ‘I still do *shisheh* when I visit some old friends; now I’m selling some used stuff which I bartered with a guy. It’s a good deal, I’m happy’. Most of the time, he repairs old watches, lighters, mobile phones and resells them in the informal markets across Tehran or to other drug users he encounters along his path. The situations in which he carries on his business comply with the ruthless rule of capital. ‘Hey *dash Reza*! What’s up? Did you make enough money from ripping me off the other day? You came and took my watch when I was lying on the ground half-dead, didn’t you?’ shouts a man while we walk in one of the parks. Reza walks straight past without paying attention to the man and explains to me, ‘he begged me to buy it, now he regrets it? *Be man ché*, why should I care?’ Drug users on a high or with heavy withdrawal symptoms can be good sellers out of euphoria or of desperate need. Reza’s mind works quickly and he is always busy doing something, whether handling some tech product he bought or calling people to set up meetings, reunions and barter sessions. His economic existence as well as that of many other pauperised drug users fits in the category of ‘jobs without definition’ (Bhatt in Denning, 2010: 89). Yet it is about work that one is speaking and not charity or theft, although stolen goods are just as good in this economy.

Chemical calibration was an expedient that Reza used to be more productive. Mobility and focus helped him not to get lost in the dregs of narcotic dependence. The practice, a *leitmotif* in my discussions with homeless users, is also common in other types of employment. Female drug users face a higher risk in the illegal market of drugs. Their bodies are an exchange product when monetary capital is absent. Hence, many sex workers make use of methamphetamine (and to a lesser extent heroin) to provide better sexual experience to their clients. It is sometimes the case that the client requires the sex worker to consume the drug in company before sexual intercourse. Meth is a powerful sexual inhibitor and it triggers sexual impulse where desire is absent or recalcitrant. Drugs help to cope with the pain and danger of sexual commerce and, in that way, sex working is likelier to pave the way to a strong addiction. *Ali Cheraq-qovveh* (Ali ‘Torch’), another young man with whom I carried out my fieldwork, discussed drugs and sex with me, while telling me about his attempts at recovery:

I learnt a lot because of my drug use, I went to many places, I met many girls, who otherwise I wouldn’t have been able to meet. They didn’t have money and were ready to give themselves in exchange for drugs, or did not have a place to use and since I had a room, they would ask me to come there to use and spend time with me. Money, I didn’t have much, but I had a place and the drugs. At the time, a lot of heroin passed through my hands and I got some cuts on it, *posht-am garm bud*, I was on the safe side. But I never dared to take advantage of these girls, I used to tell them, ‘if you don’t have money come and use with me, but don’t sell yourself, I can share it with you’. It was nice to have some female company anyway so I didn’t mind.

Mohsen’s kindness was perhaps a way to keep his reputation clean with me. But his narrative holds water:

I always kept in consideration God, even during the period when I only used drugs, when I would beg to get the money, I would still share the drugs with those who couldn’t afford it. I was a boy, I could collect rubbish, I could beg, but a girl, can she collect rubbish? Can she beg for money without risk? In these times of ours a girl who asks for money to anyone can be taken away, don’t you know?

The gendered dimension of lumpen life puts female drug users from poor backgrounds in the open market of sex and drugs. In this context, *sharing is caring* and may imply sexual concessions or friendship, whether to avoid the risk of using in public (when one’s safe space is narrow) or to extract enough capital to sustain one’s drug consumption. Sharing, however, is part of the political economy of drug use also in that it establishes lasting bonds of mutuality – at the risk of contagious diseases such as hepatitis and HIV (Bourgois, 2009). Sharing one’s drug with a companion who lacks the means to buy some or is physically impaired means that the favour might be paid back one rainy day.

The economic life of lumpen drug users is made of daily expediencies, such as barter, repairing, collecting abandoned objects, selling minimal items, begging and resorting to charity. It is a diverse ecosystem which changes according to personal and structural conditions. The use of drugs is not mechanically experienced and driven by a *deus ex machina* called ‘addiction’. It is based on what I call ‘chemical calibration’, for instance in the use of *shisheh* as a productive drug to hustling and heroin as a tranquillizer and painkiller amid sheer destitution. Philanthropy is just one side of the lumpen economy in the city.

## Epilogue

If you want to know what it means to be poor, you have to get involved and mix with the poor, if you want to know what is an addict, you have to mix with them. One doesn’t know about drugs, take him for a month to the meetings of *NA* [Narcotic Anonymous], or to a treatment camp. *Sir ta piazesho bebiné.* Mazi, you’ve got this work on the addicts, you come from Oxford, you’re cool and know all the numbers. Now you want to understand how desperate people live? You need to get destroyed in it [*khurd besham*] to understand the life of a desperate addict.

Mohsen’s incitement to immerse myself in the discourse of addiction, poverty and dangerous life was powerful methodological advice which seconded my theoretical approach ‘from below’ and my decision to go ethnographic. This perspective debunks knowledge gathered from elite interviews and epidemiological interpretation of drug abuse, which represent a good deal of studies into drug phenomena, including in Iran where epidemiology is the standard approach in drug studies. Top-down approaches, albeit articulated and linear, eventually reproduce bourgeois images and tropes, their panic, facing lumpen life. Lumpen life as seen from below reassesses the structural violence, classist xenophobia and everyday agency at work in the dangerous existences of people *in danger*.

In the first part of the article, I discussed how structural violence conditions and is conditioned by the everyday existence of homeless or precarious drug users. By letting the voice of people I carried out fieldwork with ‘speak’ about their daily occurrences, desires and expedients, I portrayed an entryway into the addiction and anti-narcotic assemblage. Imprecise, messy and disorderly, lumpen narratives – I believe – fill in the gap, with some meaning, between the attempt at theory and the empirical existences as captured though the ethnographic gaze. By showing the ecologies in which this group lives and who is part of it, the argument lingered on the structural violence of which this group is victim. This assemblage is made of their shanty dwellings – valleys, parks, graves – and the institutions of internment – prison, rehab, and morgue. Eventually, I described the economic activities that enable lumpen life by exploring the combination of agency and philanthropy. It is this panoramic view that invites the reader at an inductive approach to knowledge, which is in part a secondment of the ethnographic method that drives the analysis. On this ground, the narrative of the lumpen drug users can be revelatory of the faultlines and rationales that, *in praxis*, govern ‘addiction’ and the making of the poor into a dangerous class. Their fantasises, desires and pain – and even lies – may hold truths well beyond the word of the law and the statistical records of politico-medical agents. It might be the case that, as Michael Taussig has suggested, ‘not the basic truths, not the Being nor the ideologies of the center, but the fantasies of the marginalised concerning the secret of the center are what is most politically important to the State idea and hence State fetishism.’ (Taussig, 1992: 132).

## Notes

1. The term ‘addict’ is of problematic use and, in this article, I opt for a different terminology, where possible. I use the term ‘addict’ to report the way authorities refer to drug users and/or drug users refer to themselves. Discussion of the terminology is given ample consideration throughout the text of the article.

2. Shelter in Persian.

3. Based on the exchange rate in 2016.

4. For a historical ethnography of how drug consumption contributes to the production of space, see Ghiabi (2018d).

5. The fieldwork for this project was approved by Department of Politics and International Relations at the University of Oxford in winter 2014. Research between 2012 and 2014 was carried out via interview with officials and archival research, including over a three-month period as an intern at the United Nations Office on Drugs and Crime in Tehran. All individuals appearing in this text have been anonymised.

6. Javad is the quintessential plebeian name in Iran. Something or someone who is ‘javadi’ means ‘that belongs to a popular class’, ‘to the village’, or simply ‘poor’.

7. For a more intellectual approach to *flâneur* and narcotics, see Benjamin (1997, 2006).

8. Unpublished statistics, United Nations Office on Drugs and Crime, Tehran [Excel file].
